# The Underlying Mechanisms in the Association Between Traumatic Brain Injury in Childhood and Conduct Disorder Symptoms in Late Adolescence

**DOI:** 10.1007/s10802-022-01015-y

**Published:** 2023-01-13

**Authors:** Hanan K S. Khalaf, Alex F. Martin, Stephane A. De Brito, Edward D. Barker

**Affiliations:** 1 Department of Psychology, Department of Psychology, Institute of Psychology, Psychiatry, and Neuroscience, King’s College London, De Crespigny Park, London, SE5 8AF UK; 2 Centre for Human Brain Health, School of Psychology, University of Birmingham, Birmingham, UK

**Keywords:** Traumatic brain injury, Conduct disorder symptoms, Impulsivity, Callous unemotional traits, Family adversity, Substance use, ALSPAC

## Abstract

The present study examined i) the direct association between traumatic brain injury (TBI) in childhood and conduct disorder symptoms in adolescence, ii) whether this effect is mediated by impulsivity and/or callous unemotional traits (CU traits), and iii) whether these indirect effects are moderated by childhood family adversity and adolescent substance use. Utilising data from the Avon Longitudinal Study of Parents and Children (ALSPAC), participants with head injury information up to 12 years (4.5 years, 5.4 years, 6.5 years, 8.6 years, 11.7 years) were identified and categorised into a TBI (n = 409), orthopaedic injury (n = 1469) or non-injury group (n = 5685). Psychosocial factors such as impulsivity at 13 years, CU traits at 13 years, childhood family adversity (between birth to 4 years) and substance use at 14 years were collated for moderated mediation analyses. Conduct disorder symptoms were assessed at 16 years of age. TBI and conduct disorder symptoms were positively associated, and this association was mediated by impulsivity but not CU traits. The indirect effects were higher in magnitude for individuals with higher levels of childhood family adversity. Adolescent substance use was not found to moderate the indirect effects between TBI and conduct disorder symptoms. These results were specific to TBI individuals, and not in participants with orthopaedic injury and no reported injuries. Targeting impulsivity and early family adversity may alleviate the risk of conduct disorder symptoms following TBI in childhood. These findings have important implications for informing neuro-rehabilitative and preventative measures in clinical and community settings.

**Supplementary Information:**

The online version contains supplementary material available at 10.1007/s10802-022-01015-y.

## Introduction

Traumatic brain injury (TBI) is commonly defined as alterations in brain function, either temporarily or permanently, that are caused by external forces such as skull fractures or strikes to the head (David et al., 2010). TBI is a major health concern and can lead to numerous long-term psychiatric outcomes (Brandt et al., 2022). For example, a history of TBI in an adolescent population predicted conduct disorder alone by 5.7-fold as well as co-occurring criminality and conduct disorder by 18.7-fold (Luukkainen et al., 2012). Additionally, a meta-analysis identified a high TBI prevalence rate of 60.3% in a range of offending populations including substance use inmates and nonincarcerated offenders (Shiroma et al., 2010). These findings suggest that neurotrauma may disrupt developmental mechanisms, precipitate conduct disorder psychopathology and lead to long-term adult offending behaviours. Moreover, TBI in childhood is a common adverse event as reported by adult incarcerated populations despite being a highly preventable disease (Maas et al., 2017). Thus, TBI in childhood may be an important target for preventative interventions in mental and physical healthcare settings.

The notion that TBI may instigate conduct disorder symptoms has been suggested by several scholars (Blair, 2001; Moffitt, 2017) and numerous studies (Brandt et al., 2022; Kennedy et al., 2017). For example, Kennedy et al. (2017) utilised data from a large-scale cohort study, the Avon Longitudinal Study of Parents and Children (ALSPAC) and found that relative to children with no injuries, children with TBI were at higher risk of displaying conduct problems at 17 years. Meta-analytic evidence from structural neuroimaging data on youths with conduct disorder symptoms has also identified reduced grey matter volume in the prefrontal cortex of youths with conduct disorder, a region central for decision making and cognitive control (Rogers & De Brito, 2016). These findings are paramount as TBI in regions linked to decision making and cognitive control often lead to displays of cardinal conduct disorder symptoms like aggression or emotional dysregulation (Max et al., 2005). This has detrimental consequences to children who have sustained TBI, such as loneliness, higher likelihood for aggression and delinquency (Anderson et al., 2013), with many of these factors having been previously linked to conduct disorder (Pardini & Fite, 2010). Indeed, research has shown that children with TBI develop conduct disorder at higher rates than in a controlled population, 1-year post-injury (Gerring et al., 2009). Overall, this is suggestive of a causal association and imply that TBI may be a major risk factor for conduct disorder symptoms.

Despite the aforementioned findings, studies have yet to explore developmental processes that may help explain how TBI is related to conduct disorder symptoms. That said, there is some evidence that TBI in childhood may lead to impulsivity and callous unemotional traits (CU traits), indicating that these psychosocial factors may increase vulnerability for conduct disorder symptoms (Fanti et al., 2018). Impulsivity is commonly characterised by behaviours that are poorly conceived, risky, or inappropriate (Moeller et al., 2001), with neuroimaging evidence identifying that frontal brain injury in mice resulted in heighten long-term impulsivity, persisting 8-weeks post injury (Vonder Haar et al., 2017). Indeed, core symptoms of attention deficit hyperactivity disorder, such as impulsivity and distractibility are common sequelae of TBI in childhood (Eme, 2012). Furthermore, impulsivity has also been linked to conduct disorder symptoms such as bullying or severe rule violations (Waschbusch, 2002). CU traits are recognised as the core affective and interpersonal features of psychopathic tendencies in youth and include characteristics such as lack of empathy, guilt or remorse, shallow or deficit affect as well as a lack of concern surrounding other’s actions and feelings (De Brito et al., 2021; Viding & McCrory, 2012). Research suggests that TBI-induced lesions to the ventromedial prefrontal cortex is causally related to increased CU traits and other associated psychopathic features (i.e., impaired moral judgement; Taber-Thomas et al., 2014). Importantly, around 20% of youths with conduct disorder display elevated levels of CU traits (Centifanti et al., 2020). Although not previously explored, these findings suggest that TBI-induced impulsivity and CU traits may lead to neuropsychiatric impairments, which in turn could increase the risk for contributing to conduct disorder psychopathology.

Research also suggests that TBI may be more common in adverse family circumstances during early childhood (Guinn et al., 2019). Thus, the mediational effects of TBI-induced impulsivity and CU traits may differ depending on levels of family adversity. Family adversity is commonly characterised by factors such as harsh parental discipline, parental psychopathology, and home environment difficulties (Criss et al., 2002). Importantly, neuroimaging evidence has shown that abnormalities in the ventral striatum following exposure to early adversity are associated with deficits in motivational processes and conduct disorder development (Criss et al., 2002). Hence, these findings suggest that early family adversity may impact post-TBI functioning and conduct disorder symptoms. There is also evidence indicating that TBI-induced impulsivity and/or CU traits, which contribute to conduct disorder symptoms, may be higher for those engaging in problematic substance use (Felde et al., 2006). Substance use refers to the use of alcohol, drugs, or other psychoactive compounds (Rehm et al., 2013). There is evidence that impairments in motivation-related neural regions, such as the orbitofrontal cortex, leads to higher substance use (Dawe et al., 2004). These data suggesting that adolescents with TBI may demonstrate impaired reward-directed behaviour, which could in turn heighten substance use vulnerability. Finally, both family adversity and substance use have also been linked to greater impulsivity and youth psychopathy, which may reflect CU traits (Barker et al., 2011; Verdejo-García et al., 2008). Taken together, these findings suggest that early family adversity and adolescent substance use may each moderate the effects of impulsivity and CU traits underlying the TBI-conduct disorder symptoms association.

Utilising prospective data from the ALSPAC UK cohort, the present study is the first of its kind to examine longitudinal associations between TBI and conduct disorder symptoms to clarify if and how TBI in childhood may lead to conduct disorder symptoms in later adolescence, manifested by psychosocial factors such as impulsivity, CU traits, family adversity and substance use. It was hypothesised that TBI in childhood would be directly associated with higher levels of adolescent conduct disorder symptoms. Secondly, the TBI-conduct disorder symptom association would be mediated by impulsivity and CU traits. Finally, it was hypothesised that the association between TBI and conduct disorder symptoms would be moderated by early family adversity and adolescent substance use, such that indirect effects between TBI and conduct disorder symptoms would only be significant for individuals with high levels of family adversity and substance use.

## Methods

### Sample

The present study utilised secondary data from the birth cohort, the Avon Longitudinal Study of Parents and Children (ALSPAC; Boyd et al., 2013; Fraser et al., 2013). Initially, 14 541 pregnant women living in Avon, UK with expected delivery dates between 1st April 1991 to 31st December 1992 were invited to take part. The ALSPAC website also provides details of all the data that is available through a complete data dictionary and variable search tool (Explore data and samples | Avon Longitudinal Study of Parents and Children (https://www.bristol.ac.uk/alspac/researchers/our-data/)). Ethical approval was obtained via the ALPSAC Law and Ethics Committee and the Local Research Ethics Committees (Research ethics | Avon Longitudinal Study of Parents and Children (https://www.bristol.ac.uk/alspac/researchers/research-ethics/)). Informed consent for utilising data collected via questionnaires and clinics were acquired from participants based on recommendations of the ALSPAC Ethics and Law Committee at the time.

**Fig. 1:**
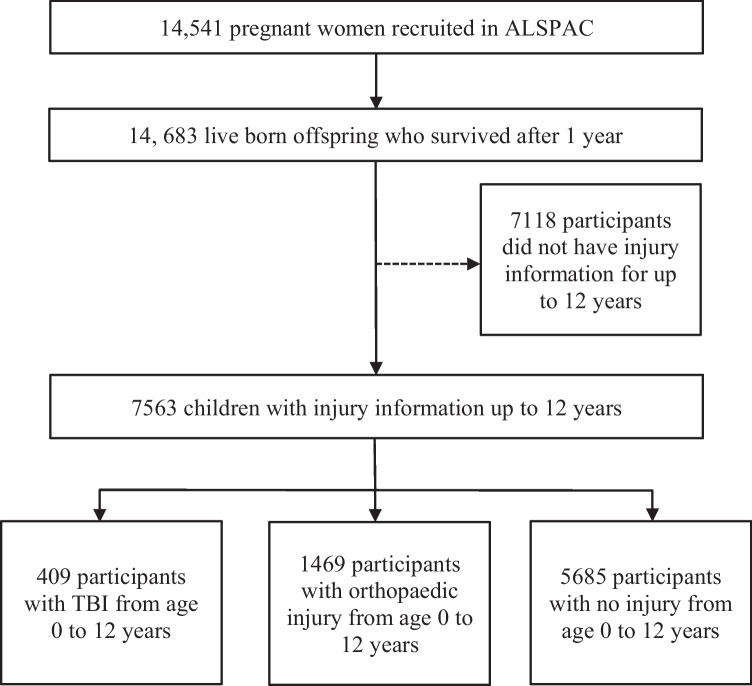
Selected sample of the study. 7118 participants were excluded as they did not have injury information from birth up to 12 years of age. *TBI* Traumatic brain injury

The current study was determined based on a sub-sample of surveyed parents with injury information relating to their children from birth up to the age of 12 years, of whom 409 had incurred a TBI, 1469 had incurred injuries not related to the skull (orthopaedic) and 5685 had no reports of injury. The final sample included 7563 participants. Those excluded were surveyed but did not respond and therefore did not provide any injury information from birth up to the age of 12 years (Fig. 1).

### Analytic Sample & Missing Data

With a large proportion of missing data (48.5%), imputation of injury information was not deemed appropriate (Little & Rubin, 2014), therefore the present study excluded any injury information where no response was provided. Excluded data was sampled with the same procedures of the included sample but consisted of participants with no responses to injury information. Therefore, participants may have information on conduct disorder symptoms, impulsivity, family adversity and other variables collected during the study but no information on their injury status.

The analytic sample was compared against those excluded using t test, chi square tests and Cohen’s d and h effect sizes as reported in Supplementary Table 1. Differences were found between groups however effect sizes were very small. Sex did significantly differ between the groups, X^2^(1, N = 14541) = 6.50, p = 0.01. Individuals in the analytic sample had lower levels of conduct disorder symptoms compared to those excluded (1.40 vs 1.50, p < 0.001, d/h = 0.11). Similarly, impulsivity (4.80 vs 4.90, p = 0.001, d/h = 0.11), CU traits (3.4 vs 3.5, p = 0.04, d/h = 0.03), and family adversity (2.4 vs 2.1, p < 0.001, d/h = 0.13) were lower in the analytic sample compared to those excluded. For substance use, individuals in the analytic sample showed greater substance use scores than those excluded (0.01 vs -0.08, p = 0.02, d/h = 0.09).

#### Injury Groups

Through multiple self-reported ALSPAC questionnaires, parents were asked if their child had incurred any injuries from birth up to the age of 12 across 5 different timepoints; 4.5 years, 5.4 years, 6.5 years, 8.6 years and 11.7 years. Timeframe of injury questions corresponded to the last timepoint at which it occurs. For example, in 4.5 years, questions included “Has been unconscious from heady injury since born” or in 11.7 years, questions included “Has broken arm or hand since 9^th^ birthday”. Participants were categorised into the TBI group if they provided a positive response to items including “head injury resulting in a loss of consciousness” or “cracked or broke skull”. Participants with positive responses to items “broke arm or hand”, “broke leg or foot” or “broken other bone” were placed in the orthopaedic injury group. Participants who had incurred a head injury as well as any other bone injuries were categorised into the TBI group. Participants with no positive reports of injuries at any time point were classed into the no injury control group. Due to the nature of questions, injury severity could not be determined and therefore, the TBI group may include children with any TBI severity.

#### Conduct Disorder Symptoms

Conduct disorder symptoms was assessed at age 16 via the Development and Well-Being Assessment (DAWBA) interview. The DAWBA is a well-validated assessment with information for the diagnosis of psychiatric disorders (Goodman et al., 2011). Examples of items include “told lies to get things or favours from others”, “often started fights” or “used a weapon or anything that could seriously hurt someone”. DAWBA was administered via a computer, with electronically generated probability bands for conduct disorder based on diagnostic criteria in the Diagnostic and Statistical Manual of Mental Disorders, 4th Edition (American Psychiatric Association, 2013; DSM-IV). Probability bands range from 0 to 5 (very unlikely to probable) such that 0: < 0.1% probability of having conduct disorder; 1: ~ 0.5%; 2: ~ 3%; 3: ~ 15%;4: ~ 50% and 5: > 70% probability.

#### Impulsivity

A 4-dimension DAWBA measure reflecting impulsivity related symptoms of attention deficit hyperactivity disorder (ADHD) was utilised. This was measured by parents when children reached the age of 13, with sum scores demonstrating an overall impulsivity score. Items in DAWBA covered the following (i) degree to which child blurts out answer before heard questions, (ii) degree to which child finds it difficult to wait their turn, (iii) degree to which often butts in other people’s conversation/games and (iv) degree to which child often goes on talking even if asked to stop/no one else listening. Sum scores ranged from 0 to 8, with higher sum scores representing greater impulsivity. DAWBA has been previously validated for the measurement of ADHD-related symptoms such as impulsivity, in children (Posserud et al., 2014).

#### Callous-unemotional Traits (CU Traits)

Callous unemotional traits (CU traits) were assessed via the ALSPAC Wellbeing of my Teenage Son/Daughter questionnaire whereby 6 items reflected CU traits at 13 years old. Items were rated on a three-point Likert scale, ranging from not true to certainly true. The following items reflect the six subscales of CU traits (Meehan et al., 2017); (i) Makes a good impression at first, but people tend to see through them after getting to know them; (ii) shallow or fast-changing emotions; (iii) usually genuinely sorry if they have hurt someone or acted badly; (iv) can seem cold-blooded or callous; (v) keeps promises; and genuine in their expression of emotions. Participants could score a total of 12 overall, with responses to “certainly true” for items 1, 2 and 4 being assigned a score of 2, “partly true” assigned a score of 1, and “not true” assigned to a score of 0. Reversed items (3, 5, 6) were scored the opposite, with “certainly true” assigned to a score of 0, “partly true” assigned to a score of 1 and “not true” assigned to a score of 2. The questionnaire has been reported to be highly associated (r = 0.81) with the callous unemotional scale of the Antisocial Process Screening Device (Meehan et al., 2017) and previously demonstrated internal reliability through confirmatory factor analysis (Barker et al., 2011).

#### Family Adversity

Indicators of family and sociodemographic risk were measured via the Family Adversity Index (FAI). The FAI was administered to parents from the birth of their child until the age of 4. The FAI is based on Rutter’s original indicators of adversity (Rutter et al., 1975) and has been previously shown to be sufficiently valid (Hardt & Rutter, 2004). The FAI covers 17 family-based risk factors across several domains including housing (i.e., inadequacy, periods of homelessness), financial difficulties, parental substance abuse (i.e., drugs, alcohol), parental psychopathology (i.e., depression, anxiety), and involvement with crime (i.e., convictions, trouble with police). If adversity is present in in each domain, a rating of 1 was obtained. Total FAI scores were gained by summing all risk factors, with higher scores indicating higher adversity.

#### Substance Use

Substance use information was collected from children when they were 14 years via an interview as part of the ALSPAC clinic assessment. Questions of the interview were adapted from the validated adolescent version of the Semi-Structured Assessment for the Genetics of Alcoholism (SSAGA; Acion et al., 2019; Bucholz et al., 1994). SSAGA questions as part of the ALSPAC interview examined substance use patterns. The present study utilised 3 items measuring the frequency of engagement with alcohol, cigarette smoking and cannabis use over 6 months prior to the interview being administered. SSAGA questions were adapted by the ALSPAC team with the intention to build upon findings related to maladaptive substance use. The adaptation for each item was that the question (related to cigarette smoking, alcohol or cannabis use) asked *how many times in the last 6 months* compared to the original item which asked about the *frequency of substance use per day and the duration of substance use in months.* Additionally, the response was coded into categories by the ALSPAC team (Nil, 1 per week, 1–3 times, > 4 times). The researchers did not make any additional adaptations for this study. The items and the associated SSAGA items are reported in full in Supplementary Table 2. Scores were included in a principal component analysis with varimax rotation to obtain an overall composite score reflecting substance use. A principal component analysis is a multivariate exploratory analysis method that reduces multidimensional data while retaining depth of information (Karamizadeh et al., 2013). Thus, it was deemed appropriate to extract a substance use dimension employing this framework. The analysis yielded one component with an eigenvalue of 1.51, explaining 50.4% of the variance, which is deemed acceptable (Houle et al., 2002).

#### Confounders

Variables that have been previously established to be associated with TBI and conduct disorder but not deemed as part of the causal pathway, were identified as confounders, and incorporated in the analysis as such. These include, sex, conduct disorder symptoms at 8 years, impulsivity at 8 years, intelligence, harsh parenting and family adversity (Guinn et al., 2019; Königs et al., 2016; Schorr et al., 2020). Family adversity was included as a confounder through the FAI measure when it was not acting as a moderator of mediation. Specifically, apart from Model 3, where moderation effects of family adversity were tested, the FAI measure was incorporated as a confounder. Information on sex was obtained from the ALSPAC child baseline sample data. Akin to conduct disorder symptoms at 16 years, DAWBA was administered via interviews to obtain computer generated diagnostic bands for conduct disorder at 8 years based on the DSM-IV. The Wechsler Intelligence Scale for Children, Third Edition (WISC-III) was administered to participants when they were 9 years old to obtain an overall intelligence quotient (IQ) score. The WISC-III has been previously shown to be highly reliable and valid when measuring intelligence in children (Allen et al., 2014). Moreover, WISC-III provides scores (M = 100, SD = 15), therefore, WISC-III scores were standardised via z-score standardisation to ensure scores were compatible with other variables for analysis. Score ranged between -3.56 to 2.84, with higher scores indicating higher IQ scores. Akin to impulsivity at 13 years, DAWBA measuring impulsivity related symptoms of ADHD at 8 years was utilised. Harsh parenting was assessed at 2 years by parents responding to questions such as ‘When at home with your child, how often do you do the following”: 1) shout at him/her and/or 2) slap her/him. Responses were collected on a scale (reversed coded) from 1 (everyday) to 5 (rarely/never).

## Data Analysis

**Fig. 2:**
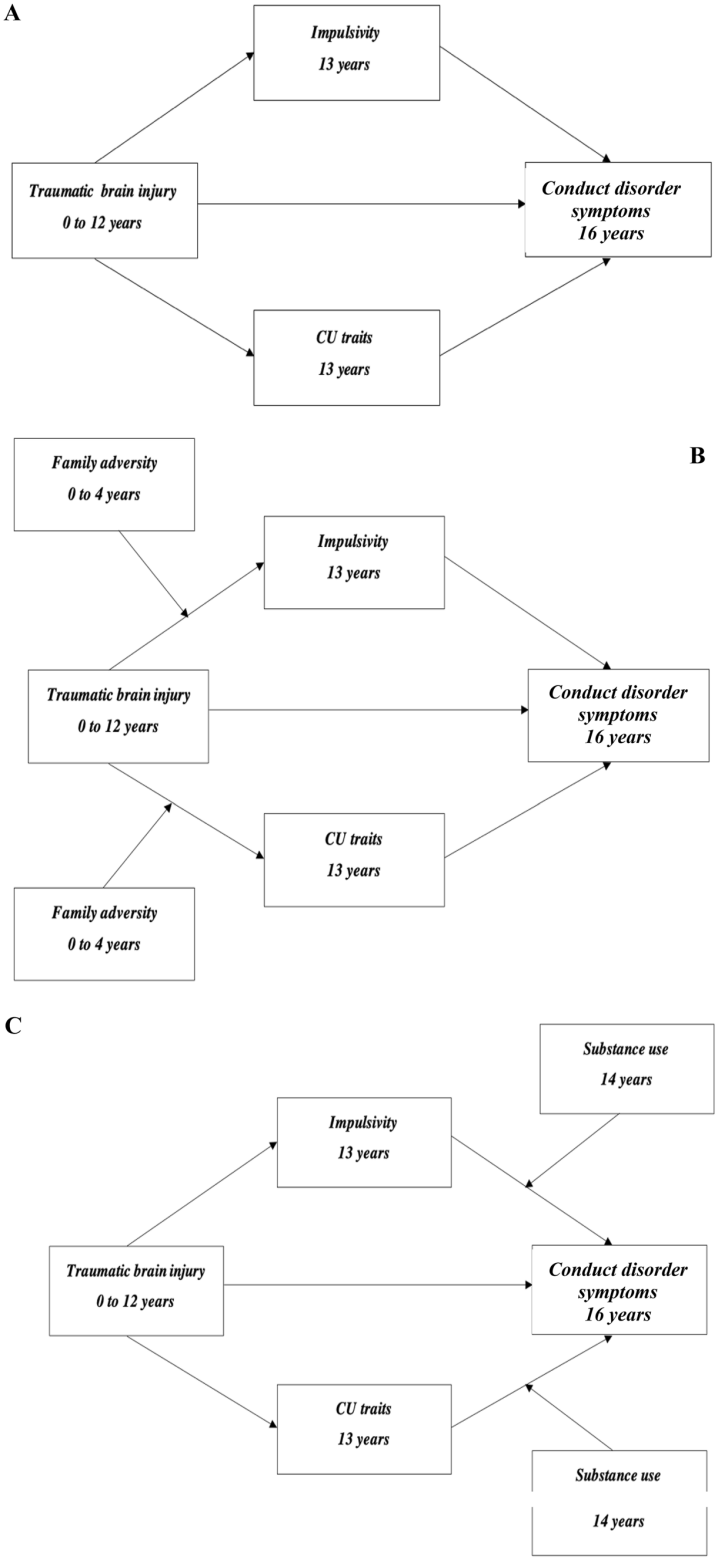
Moderated mediation models (**A**) Model 2: Mediation model, testing the hypothesis wherein the presence of impulsivity and callous unemotional traits would mediate the relationship between TBI and conduct disorder symptoms. **B** Model 3: Family adversity moderation model, testing hypothesis wherein the presence of family adversity would moderate the strength of indirect effects between TBI and conduct disorder symptoms. **C** Model 4: Substance use moderation model, testing hypothesis wherein the presence of substance use would moderate the indirect effects between TBI and conduct disorder symptoms. CU traits = Callous unemotional traits

In the main analysis, participants with TBI were compared to uninjured participants in moderated-mediation models. Thus, the final sample for the main analysis includes children with TBI (n = 409) and uninjured individuals (n = 5685). As part of the moderated mediation models, mediator and moderator effects are combined, wherein the indirect effect of the predictor on outcome, varies depending on the levels of the moderator (Miles et al., 2015). The analysis proceeded in the following steps; Firstly, the relationship between TBI and conduct disorder symptoms was examined. Secondly, the mediating role of impulsivity and CU traits was examined (Model 2; Fig. 2). Nonsignificant pathways were identified, removed, and subsequent addition of family adversity was made into the model as a moderator on the pathways linking TBI and significant mediators (Model 3; Fig. 2). Following this, substance use was included as a moderator on pathways linking significant mediators to conduct disorder symptoms (Model 4; Fig. 2). Sex, intelligence, conduct disorder symptoms at 8 years, impulsivity at age 8 and harsh parenting at age 2 were included as confounders throughout the analysis. Family adversity was additionally included as a confounder when it did not act as a moderator to account for early adverse effects.

R studio (RStudio. Rstudio.com, n.d.) Version 1.3.1093 was used to conduct all statistical analyses. Bivariate correlations were conducted to examine the relationships between variables. The 95% confidence intervals (CI) generated by percentile bootstrapping with a resample rate of 10,000 was utilised to assess reliability of model effects and avoid inflation of type I error rate. Bootstrapping is a nonparametric computational resampling technique which considers skewed standard errors underlying indirect effects (Biesanz et al., 2010). Percentage of the total effects that was accounted for by indirect effects were obtained through the R^2^ value. The 95% CI for unstandardised indirect effects were determined at the 2.5th and 97.5th percentiles, such that if it did not carry zero at a level of confidence (p < 0.05), the results were considered consistent with the study’s hypothesis (Biesanz et al., 2010).

### Sensitivity Analyses

Additional moderated mediation models were employed to compare models from the main analysis. Firstly, an orthopaedic model (Model 5) including orthopaedic individuals and uninjured individuals was computed. Secondly, a model consisting of individuals with TBI and individuals with orthopaedic injury (Model 6) was computed and results of both models were compared with the main analysis to identify whether results were specific to the TBI group.

## Results

Table 1 shows the mean and standard deviation of key variables. Within the sample, 5.41% represented the TBI group, 19.42% represented the orthopaedic group and 75.15% represented the non-injured group. These percentages match closely with previous proportions based on existing epidemiological data within ALSPAC (Kennedy et al., 2017).

**Table 1:** Mean and standard deviations of key variables

		***Variables***									
		Primary variables					Covariates				
		Conduct disorder symptoms	Family adversity	Substance use	Callous unemotional traits	Impulsivity	Sex	Intelligence	Conduct disorder symptoms	Impulsivity	Harsh Parenting
		age 16	birth to age 4	age 13	age 13	age 13	(% male)	age 9	age 8	age 8	age 2
**TBI** **(n = 409; 5.41%)**		1.50 (0.70)	3.13 (2.89)	0.05 (1.07)	3.92 (2.16)	5.21 (2.08)	59.0	-0.09 (1.1)	1.57 (.59)	5.20 (1.80)	6.00 (1.70)
**Orthopaedic (n = 1469; 19.42%)**		1.38 (0.59)	2.44 (2.62)	0.03 (1.02)	3.48 (1.97)	4.80 (1.58)	50.0	0.06 (.97)	1.42 (.59)	5.20 (1.80)	5.80 (1.66)
**No injury** **(n = 5685; 75.15%)**		1.36 (0.64)	2.39 (2.50)	-0.03 (.97)	3.48 (1.97)	4.72 (1.47)	49.0	0.09 (.98)	1.41 (.55)	5.10 (1.70)	5.90 (1.70)

Conduct disorder symptoms were assessed via the Developmental and Well-being Assessment (DAWBA) interview. Scores ranged between 0 and 5, with 5 being the highest probability (> 70%) of developing conduct disorder symptoms. Family adversity were assessed via the Family Adversity Index (FAI). Scores ranged from 0 to 20, with higher scores indicating higher adversity. Substance use was assessed via the adapted version Structured Assessment for the Genetics of Alcoholism. Scores were included in a principal component analysis with varimax rotation to obtain an overall composite score reflecting substance use. Callous unemotional traits (CU traits) were assessed via the ALSPAC Wellbeing of my Teenage Son/Daughter questionnaire whereby certain items reflect the six subscale of CU traits. Scores ranged from 0 to 12 with higher scores indicating higher levels of CU traits. Impulsivity were assessed via DAWBA. Scores ranged from 0 to 8 with higher scores reflecting higher impulsivity. Information on sex was obtained from the ALSPAC child baseline sample data. Scores on intelligence were obtained from the Wechsler Intelligence Scale for Children, Third Edition (WISC-III). WISC-III scores were standardised via z-score standardisation to ensure scores were compatible with other variables for analysis. Thus, score ranged between—3.56 to 2.84, with higher scores indicating higher intelligence. Harsh parenting was assessed via parents reported responses to questions related verbal (shouting) and physical (slapping) parenting. Scores ranged from 1 to 5, with higher scores indicating higher levels of harsh parenting

*TBI* Traumatic brain injury

Table 2 shows the bivariate Pearson’s correlations for all study variables. TBI was positively correlated to conduct disorder symptoms, CU traits, impulsivity, and family adversity but not substance use. TBI was also positively correlated to conduct disorder symptoms at age 8, impulsivity at age 8, negatively correlated to intelligence at age 9 and not correlated to harsh parenting. Conduct disorder symptoms was positively correlated to substance use and negatively correlated to intelligence.

**Table 2:** Bivariate Pearson’s correlations of key variables

	**TBI**	**Ortho**	**CU traits**	**Intelligence**	**Impulsivity**	**FAI**	**CD (age 16)**	**CD (age 8)**	**Impulsivity (age 8)**	**Harsh parenting (age 2)**
**TBI (n = 409)**										
**Orthopedic (n = 1469)**										
**CU traits (age 13)**	0.06**	0.01								
**Intelligence (age 9)**	-0.04**	-0.01	-0.09**							
**Impulsivity (age 13)**	0.07**	0.02	0.34**	-0.12**						
**Family adversity (FAI; birth to 4 years)**	0.07**	0.00	0.15**	-0.14**	0.16**					
**CD (age 16)**	0.05**	0.03	0.34**	-0.12**	0.24**	0.20**				
**CD (age 8)**	0.07**	0.00	0.25**	-0.09**	0.25**	0.19**	0.23**			
**Impulsivity (age 8)**	0.06**	0.03	0.28**	-0.13**	0.52**	0.18**	0.21**	0.34**		
**Harsh parenting (age 2)**	0.02	0.04	0.16**	-0.10	0.11**	0.08**	0.12**	0.15**	0.16**	
**Substance use (age 13)**	-0.02	-0.03*	-0.11**	-0.01	0.06**	0.09**	0.20**	0.08**	-0.06**	-0.02

TBI and orthopaedic injury were grouped with the no injury control participants (n = 5687) to create a dichotomous variable and produce the bivariate correlations

*M* Mean, *SD* Standard deviation, *TBI* Traumatic brain injury, *CU traits* CU traits, *CD* Conduct disorder symptoms, *FAI* Family adversity index, *SU* Substance use

*p value < 0.05; **p value < 0.00

### Direct Effects

The presence of TBI in childhood was found to lead to higher levels of conduct disorder symptoms at 16 years (β = 0.087, *p* < 0.05; Table 3).

**Table 3:** Results of model 2 mediation model

	**β**	**SE**	**p value**	**95% CI**	
				**LL**	**UL**
**Direct effects**					
TBI → CD	0.087	0.057	0.039	0.024	0.14
**Indirect effects**					
TBI → impulsivity → CD	0.030	0.015	0.043	0.005	0.063
TBI → CU traits → CD	0.021	0.014	0.115	-0.004	0.049
**Total effects**					
TBI → CD	0.140	0.049	0.004	0.045	0.240

Confounders include sex, intelligence, early conduct disorder, early impulsivity, family adversity and harsh parenting

*TBI* Traumatic brain injury, *CD* Conduct disorder symptoms, *CU traits* Callous unemotional traits, *β* unstandardized coefficient, *SE* standard error, *CI* confidence interval, *LL* Lower limit, *UL* Upper limit

### Model 2- Mediation Model

**Fig. 3:**
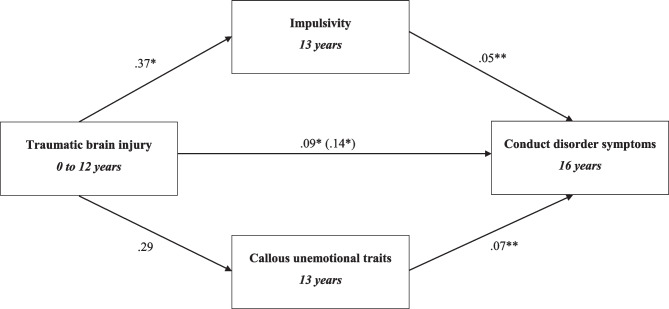
Model 2: Mediation model, testing hypothesis A wherein the presence of impulsivity and callous unemotional traits would mediate the relationship between TBI and conduct disorder symptoms. Unstandardised indirect effects are presented. The effects on the direct path from traumatic brain injury to conduct disorder symptoms depict the direct effect and the (total effect). Confounders in this model include sex, intelligence, family adversity, early conduct disorder symptoms (at age 8), early impulsivity (age 8) and harsh parenting. *p value < 0.05, **p value < 0.00

Model 2 tested the mediating role of impulsivity and CU traits in the relationship between TBI and conduct disorder symptoms (Fig. 3). The mediation model included the following controls: Intelligence, conduct disorder at age 8, sex, impulsivity at age 8, harsh parenting and family adversity. As seen in Table 2, unstandardised indirect effects were computed for each of 10,000 bootstrapped samples, which returned 0.030 (CI, 0.005, 0.063) for impulsivity and 0.021 (CI, -0.004, 0.049) for CU traits (Table 3). These indirect effects accounted for 26% and 29% of the total effects respectively. As CU traits were not found to mediate the relationship between TBI and conduct disorder symptoms, it was not explored further in the following models. As the 95% CI for impulsivity did not carry zero at a level of confidence (p < 0.05), the indirect effect was considered worth further investigation. Impulsivity was found to mediate the long-term relationship between TBI and conduct disorder symptoms.

**Fig. 4:**
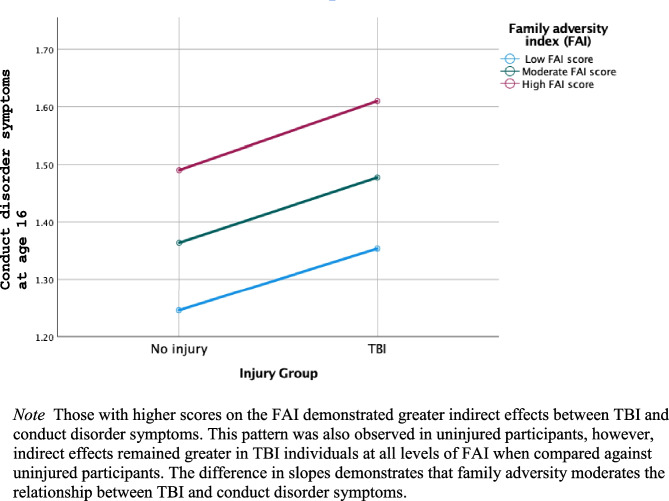
Moderation effects of family adversity on the relationship between TBI and conduct disorder symptoms

**Fig. 5:**
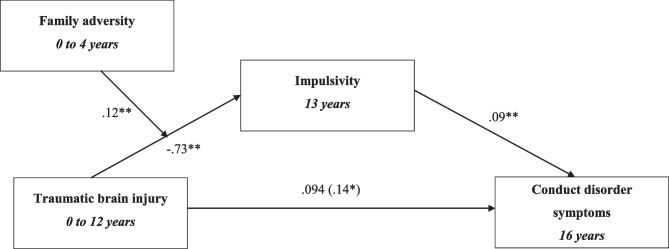
Model 3: Family adversity moderation model, testing hypothesis B whereby the presence of family adversity would moderate the strength of indirect effects between TBI and conduct disorder. Unstandardised indirect effects are presented. The effects on the direct path from traumatic brain injury to conduct disorder depict the direct effect and the (total effect). Confounders in this model include sex, intelligence and early conduct disorder symptoms (at age 8), early impulsivity (age 8) and harsh parenting. *p value < 0.05, **p value < 0.00

### Model 3- Family Adversity Moderation Model

Model 3 examined whether early family adversity would moderate the relationship between TBI and conduct disorder symptoms (Fig. 4). The moderation of the indirect effects is plotted in Fig. 5. The unstandardised conditional indirect effect for impulsivity was found to be 0.120 (95% CI, 0.106, 0.150; Table 4). The conditional indirect effects for participants 1 SD below and above the mean of the FAI returned 0.110 (95% CI, -0.001, 0.230) and 0.120 (95% CI, 0.001, 0.230) respectively. At the mean level, the conditional indirect effect was 0.120 (95% CI, 0.017, 0.220). Thus, family adversity was found to moderate the relationship between TBI and conduct disorder symptoms, such that the indirect effect through impulsivity is larger for individuals with higher levels of family adversity (Fig. 5).

**Table 4:** Results of model 3: Family adversity moderation model

	**β**	**SE**	**p value**	**95% CI**	
				**LL**	**UL**
**Direct effects**					
TBI → CD	0.094	0.059	0.11	-0.017	0.210
**Indirect effects**					
TBI → impulsivity → CD	0.026	0.013	0.025	0.004	0.053
TBI x FAI → impulsivity	0.120	0.013	0.000	0.106	0.150
**Total effects**					
TBI → CD	0.140	0.049	0.004	0.045	0.240
**Conditional indirect effects at different FAI values**					
1 SD below mean FAI	0.110	0.059	0.051	-0.001	0.230
Mean FAI	0.120	0.051	0.022	0.017	0.220
1 SD above mean FAI	0.120	0.055	0.027	0.001	0.230

Confounders include sex, intelligence, early conduct disorder symptoms, early impulsivity, and harsh parenting

*TBI* Traumatic brain injury, *CD* Conduct disorder symptoms, *β* Unstandardized coefficient, *SE* Standard error, *CI* confidence interval, *LL* Lower limit, *UL* Upper limit, *FAI* Family adversity index, *SD* Standard deviation

### Model 4- Substance Use Moderation Model

**Fig. 6:**
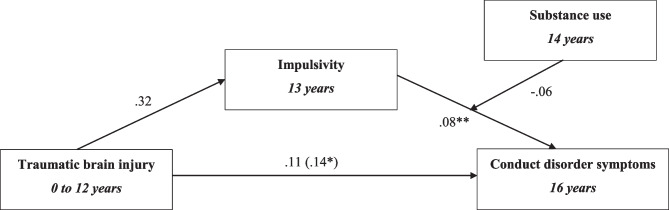
Model 4: Substance use moderation model, testing hypothesis B wherein the presence of substance use would moderate the relationship between TBI and conduct disorder symptoms. Unstandardised indirect effects are presented. The effects on the direct path from traumatic brain injury to conduct disorder symptoms depict the direct effect and the (total effect). Confounders in this model include sex, intelligence, family adversity and early conduct disorder symptoms (at age 8), early impulsivity (age 8) and harsh parenting. *p value < 0.05, **p value < 0.00

Model 4 examined the hypothesis that substance use would moderate the relationship between TBI and conduct disorder symptoms (Fig. 6). The indirect effects of impulsivity returned -0.025 (95% CI, 0.00, 0.057). The conditional indirect effect of substance use for participants below and above the mean returned 0.093 (95% CI, -0.047, 0.230) and 0.028 (95 CI, -0.277, 0.330) respectively. The conditional indirect effect at mean level of substance use was 0.060 (95% CI, -0.157, 0.280). Thus, as the conditional indirect effects carried zero within the 95% CI, substance use was not found to significantly moderate the association between TBI and conduct disorder symptoms (Table 5).

**Table 5:** Results of model 4: Substance use moderation model

	**β**	**SE**	**p value**	**95% CI**	
				**LL**	**UL**
**Direct effects**					
TBI → CD	0.011	0.059	0.073	-0.009	0.230
**Indirect effects**					
TBI → impulsivity → CD	0.025	0.015	0.009	0.000	0.057
Impulsivity x SU → CD	-0.009	0.012	0.460	-0.036	0.012
**Total effects**					
TBI → CD	0.140	0.049	0.004	0.045	0.250
**Conditional indirect effects at different SU values**					
1 SD below mean SU	0.093	0.071	0.019	-0.047	0.230
Mean SU	0.060	0.111	0.590	-0.157	0.280
1 SD above mean SU	0.028	0.155	0.860	-0.277	0.330

Confounders include sex, intelligence, early conduct disorder symptoms, early impulsivity, family adversity and harsh parenting

*TBI* Traumatic brain injury, *CD* Conduct disorder symptoms, *β* Unstandardized coefficient, *SE* Standard error, *CI* confidence interval, *LL* Lower limit, *UL* Upper limit, *SU* Substance use, *SD* Standard deviation

### Sensitivity Analysis

Model 5: Orthopaedic sensitivity model, included orthopaedic individuals (n = 1469) and uninjured individuals (n = 5685). The orthopaedic model returned an indirect impulsivity effect of 0.008 (95% CI, 0.000, 0.015). As the indirect effect contained zero within the 95% CI, impulsivity was not found to mediate the relationship between orthopaedically injured individuals and conduct disorder symptoms (Table 6). Moreover, below and above mean level of the conditional indirect effects of family adversity was found to be 0.002 (95% CI, -0.052, 0.009) and 0.017 (95% CI, -0.055, 0.090) respectively. The mean conditional indirect effect of family adversity also returned 0.018 (95% CI, -0.032, 0.068). These findings suggests that family adversity did not significantly moderate the relationship between orthopaedically injured participants and conduct disorder symptoms. Thus, results of the main analysis were found to be specific to the TBI group (Table 7).

**Table 6:** Model 5; Orthopaedic sensitivity model, with orthopaedically injured participants and uninjured participants

	**β**	**SE**	**p value**	**95% CI**	
				**LL**	**UL**
**Direct effects**					
OI → CD	0.037	0.027	0.170	-0.016	0.093
**Indirect effects**					
OI → impulsivity → CD	0.008	0.003	0.061	0.000	0.015
OI x FAI → impulsivity	0.130	0.012	0.000	0.111	0.160
**Total effects**					
OI CD	0.018	0.025	0.490	-0.032	0.067
**Conditional indirect effects at different FAI values**					
1 SD below mean FAI	0.002	0.036	0.060	-0.052	0.009
Mean FAI	0.018	0.025	0.480	-0.032	0.068
1 SD above mean FAI	0.017	0.037	0.640	-0.055	0.090

Confounders include sex, intelligence, early conduct disorder symptoms, early impulsivity, and harsh parenting

*OI* Orthopaedic injury, *CD* Conduct disorder symptoms, *β* Unstandardized coefficient, *SE* Standard error, *CI* confidence interval, *LL* Lower limit, *UL* Upper limit, *FAI* Family adversity index, *SD* Standard deviation

**Table 7:** Model 6; TBI vs Orthopaedic sensitivity model, with TBI participants and orthopaedically injured participants

	**β**	**SE**	**p value**	**95% CI**	
				**LL**	**UL**
**Direct effects**					
TBI → CD	0.063	0.061	0.300	-0.056	0.180
**Indirect effects**					
TBI impulsivity → CD	0.002	0.023	0.050	0.002	0.043
TBI x FAI → impulsivity	0.170	0.019	0.000	0.140	0.210
**Total effects**					
TBI → CD	0.018	0.025	0.490	-0.032	0.067
**Conditional indirect effects at different FAI values**					
1 SD below mean FAI	0.093	0.074	0.021	-0.005	0.240
Mean FAI	0.099	0.052	0.050	0.002	0.020
1 SD above mean FAI	0.011	0.065	0.050	0.022	0.023

Confounders include sex, intelligence, early conduct disorder symptoms, early impulsivity, and harsh parenting

*TBI* Traumatic brain injury, *CD* Conduct disorder symptoms, *β* Unstandardized coefficient, *SE* Standard error, *CI* confidence interval, *LL* Lower limit, *UL* Upper limit, *FAI* Family adversity index, *SD* Standard deviation

Model 6: TBI vs Orthopedic sensitivity model, included individuals with TBI (n = 409) and individuals with orthopedic injury (n = 1469). Model 6 returned an indirect impulsivity effect of 0.002 (95% CI, 0.002, 0.043). Moreover, below and above the mean leave of the conditional indirect effects of family adversity returned 0.093 (95% CI, -0.005, 0.240) and 0.011 (95% CI, 0.022, 0.023) respectively. The mean conditional indirect effects of family adversity returned 0.099 (95% CI, 0.002, 0.020). Thus, impulsivity and family adversity were found to be a significant mediator and moderator to the TBI- conduct disorder symptom association respectively. Results of the main analysis are therefore specific to the TBI group.

#### Discussion

Employing the epidemiological ALSPAC cohort, the present study examined whether impulsivity and CU traits might help explain how TBI in childhood associates with conduct disorder symptoms in adolescence. Further, we tested whether family adversity and substance use would moderate any indirect associated found. For example, is that children exposed to higher levels of family adversity are at risk for TBI, and that TBI then increases impulsivity or CU traits, which in turn heightens vulnerability for conduct disorder symptoms. In line with our hypothesis, TBI in childhood was directly associated with conduct disorder symptoms at 16 years. Furthermore, impulsivity alone was found to significantly mediate the association between TBI and conduct disorder symptoms. Specifically, children with TBI were more likely to demonstrate higher levels of impulsivity, which in turn, increased risk for conduct disorder symptoms in adolescence. In terms of moderating effects, partially supporting our hypothesis, the impulsivity mediational pathway was greater in magnitude for youths who were exposed to higher levels of family adversity. However, a moderational pattern was not identified for substance use, suggesting that the mediational pathways are not higher for youths who engage in higher levels of substance use at age 14.

The present study is the first of its kind to identify impulsivity as a potential mechanism explaining why children with TBI are subsequently more vulnerable to develop conduct disorder symptoms. Indeed, damage due to TBI can lead to numerous neuropsychiatric sequelae such as impaired attention and increased impulsivity (Bechara & van der Linden, 2005). Impulsivity can subsequently lead to increased sensation seeking, aggression and poor decision making (Moeller et al., 2001), all of which are observed in conduct disorder psychopathology (Fairchild et al., 2019; Fanti et al., 2018). Indeed, Kagan’s behavioural theory of impulsivity postulates that impulsivity influences behavioural and cognitive responses to situations and can enable behaviours are dangerous, inappropriate or have negative consequences (Kagan et al., 2018). In this context, our findings thus have theoretical implications as they support past research findings but also crucially extend them by providing a unified and mechanistic longitudinal understanding of how impulsivity may explain the TBI-conduct disorder symptom association.

In line with past evidence (Boes et al., 2011; Taber-Thomas et al., 2014), TBI and CU traits were positively correlated in that the presence of TBI was associated with higher levels of CU traits. However, the present study did not find a mediation effect on CU traits between TBI and conduct disorder symptoms. A potential reason for this can explained by neurocognitive models of the development of psychopathic/CU traits (Blair, 2013), which implicate the amygdala as the main substrate for the emotional impairments characterising CU traits, whereas common neural regions associated with TBI such as the ventromedial prefrontal cortex (Boes et al., 2011; Taber-Thomas et al., 2014), are more related to poor decision making and impulsivity. Thus, elevated CU traits are not likely a result of TBI, due to TBI-induced changes being more commonly associated with cortical rather than subcortical lesions (Blair, 2001). Moreover, only a minority of adolescents with conduct disorder symptoms display elevated levels of CU traits (Centifanti et al., 2020), while impulsivity is more commonly observed in various forms of conduct disorder presentations (Waschbusch, 2002). It is also important to consider that CU traits may not have a mediating effect on the present study’s sample of individuals with TBI. Past research suggests neuropsychiatric symptoms such as aggression following TBI to be linked to TBI severity (Rao et al., 2009). Hence, the mediating role of CU traits in the TBI-conduct disorder symptom association may only be present in a subsample of moderate to severe individuals with TBI, something the present study could not account for due to the absence of TBI severity information. Thus, future research should be conducted in more high-risk samples. Indeed, understanding CU traits in the TBI-conduct disorder symptom association can inform theories surrounding CU traits and inappropriate behaviour (Pisano et al., 2017; Roose et al., 2011), but also to promote preventative measures and early intervention.

Another important finding in the present study is family adversity as a significant moderator of the impulsivity mediational pathway. Specifically, the indirect effects of impulsivity were higher in magnitude for children who experienced higher levels of early family adversity. Consistently, research shows that adverse family experiences such as abuse or parental psychopathology, can lead to greater risk of developing lifetime conduct disorder symptoms, with risk increasing as exposure to adversity heightens (Green et al., 2010). Furthermore, common factors associated with early family adversity such as neglect or exposure to violence can increase vulnerability to TBI (Criss et al., 2002). Family adversity can subsequently impact development of psychiatric conditions through TBI incidences (Gerry Taylor et al., 2001; Guinn et al., 2019; Jackson et al., 2022). Specifically, Jackson et al. (2022) revealed that conduct problems explained approximately 23% of the association between youth adversity and TBI, thus indicating that TBI risk is increased with youth adversity which in turn heighten risk for psychiatric consequences. Thus, our findings provide a transactional view of the TBI-impulsivity-conduct disorder symptom association by highlighting the role of early family adversity in child injury and the increased risk to psychopathology in late adolescence.

Although the present study identified family adversity as a significant moderator between TBI and conduct disorder, substance use was not found to influence this association. A potential explanation may be due to the time point and nature at which substance use datum were collected. Specifically, the present study collected substance use data through interview sessions when participants were 14 years old, an age determined as past literature identified early onset substance use as a major risk factor in developing substance use dependency in later life (Morean et al., 2014). However, engaging with substances at this age is considered rare, with research reporting the median age of alcohol and cigarette use between 16 to 21 years while illicit drugs are reported between 18 to 24 years old (Degenhardt et al., 2016). Thus, the minority of children who engage with substances may be less incline to report this, particularly during interview questioning, as it is not the social norm at 14 years old. Overall, future research is warranted to elucidate the role of substance use underlying the TBI-conduct disorder symptom association.

Several limitations of the present study are noteworthy. Firstly, due to the aggregated nature of the TBI variable, detailed information was not obtained such as TBI severity, location, exact age of injury or number of TBI incidences. Although aggregating TBI across 5 different timepoints allowed for a measure across childhood, it is also possible that certain injury incidences may only occur at the last timepoint of 11.7 years. This limits the time to capture conduct disorder symptoms at age 16 by only 4 years. Moreover, it is also possible that TBI may be sustained outside the timepoints captured i.e., between 11.7 years to 16 years, something which could not be documented in the present study. Thus, results of the present study should be interpreted with caution. Secondly, impulsivity is multifaceted with aspects such as behavioural impulsivity (i.e., response inhibition) and cognitive impulsivity (i.e., inability to delay satisfaction; Bakhshani, 2014). Thus, although a positive mediational effect was identified, it remains unclear which aspect of impulsivity is most important to the TBI-conduct disorder symptom association.

The longitudinal nature of ALSPAC also encountered problems with attrition. For example, over 9000 responses were collated on injury information at the first time point of 4.5 years. However, the final subsample of injury response decreased to approximately 7500 participants, suggesting a 21.2% drop-out rate on injury information. Moreover, there was a difference in sex between the analytic sample and those excluded as well as in scores in conduct disorder symptoms, impulsivity, CU traits, family adversity and substance use. Wolke et al. (2009) noted that individuals with disruptive behaviours within ALSPAC are more frequently lost to follow up. Indeed, Brame and Piquero (2003) noted that individuals with higher levels of delinquency are more likely to drop out of longitudinal studies. Thus, observing changes to development and delinquent-related behaviours may be negatively biased due to the lack of higher scoring participants. Moreover, It is also important to note that the correlation between TBI and conduct disorder symptoms is relatively small (*r* = 0.05, p < 0.00), and could be a function of the large sample size of the current study. Overall, results of the present study should be interpreted with caution.

Despite these limitations, the ALSPAC cohort design still presented with strengths such as the inclusion of early conduct disorder effects as a confounder. As conduct disorder in early life increases the risk of subsequently developing TBI (Bandyopadhyay et al., 2020), controlling for early conduct disorder effects allowed the present study to determine that conduct disorder symptoms in late adolescence was indeed due TBI during childhood. This is particularly important when exploring underlying mechanisms of a developmental pathway (Kwok et al., 2008). Indeed, results demonstrated that impulsivity was a significant mediator in the relationship between TBI and conduct disorder symptoms.

It is also known that orthopedically injured participants have similar confounding structures to individuals with TBI with no biological connection to conduct disorder (Kwok et al., 2008). Therefore, the inclusion of negative control groups such as orthopaedically injured participants and uninjured participants allowed for the deduction of whether results were specific to children with TBI. Sensitivity analyses from the present study demonstrated that orthopaedic injury did not lead to higher risk of developing conduct disorder symptoms, with impulsivity and family adversity not found to mediate and moderate this relationship. Furthermore, when children with TBI were compared against those with orthopedic injury, the association between injury and outcomes of interests remain significant. Thus, findings from the sensitivity analysis allowed the present study to conclude that indirect effects identified were specific to TBI individuals.

Overall, impulsivity and early family adversity are important psychosocial factors that lead to higher risk of conduct disorder symptoms following TBI in childhood. These findings are paramount in informing clinicians on tailoring preventative and neuro-rehabilitative measures to offset any risk of conduct disorder psychopathology and potential crimes. For example, these results suggest that early screening for impulsivity when children present to health services following TBI incidences or intervening family adversity through social programs may benefit and become a protective factor for conduct disorder psychopathology. These findings also corroborate previous research findings which suggest that early interventions can prevent adult psychopathology among high-risk children with conduct problems (Dodge et al., 2015). Moreover, a history of TBI may contribute to an offender’s behaviours and arrest, although this is rarely considered for a high number of youths under custody (Hughes et al., 2015). Thus, findings of the present study are critical to inform appropriate services and promote rehabilitative interventions for this population.

## Supplementary Information

Below is the link to the electronic supplementary material.

Supplementary file1 (DOCX 25 KB)
