# Supplementary material for: The Underlying Mechanisms in the Association Between Traumatic Brain Injury in Childhood and Conduct Disorder Symptoms in Late Adolescence
Source: Res Child Adolesc Psychopathol. 2023 Jan 13;51(5):709–25. doi: 10.1007/s10802-022-01015-y (PMC10119055; doi:10.1007/s10802-022-01015-y)
Supplement: Supplementary file 1 — Supplementary file1 (DOCX 25 KB) [file 10802_2022_1015_MOESM1_ESM.docx]

| **Supplementary Table 1.** Comparisons between the analytic sample and the excluded sample | | | | | | | | | | | | | | |
| --- | --- | --- | --- | --- | --- | --- | --- | --- | --- | --- | --- | --- | --- | --- |
|  |  |  | **Analytic sample (N = 7565)** | | |  | **Excluded sample (N = 6976)** | | |  | **Difference test and effect size** | | | |
|  |  |  | Mean | *SD* | Range |  | Mean | *SD* | Range |  | *t/ chi^2^* | *df* | *p* | *d / h* |
| Conduct disorder symptoms  Impulsivity  CU traits  Family adversity  Substance use | | | 1.40 | 0.64 | 0-5 |  | 1.50 | 0.75 | 0-5 |  | 4.00 | 714 | < .001 | 0.11 |
|  |  |  | 4.8 | 1.52 | 0-12 |  | 4.9 | 1.78 | 0-12 |  | 3.00 | 963 | 0.01 | 0.11 |
|  |  |  | 3.4 | 2.00 | 0-12 |  | 3.5 | 2.03 | 0-12 |  | 0.80 | 995 | 0.04 | 0.03 |
|  |  |  | 2.4 | 2.55 | 0-20 |  | 2.1 | 2.61 | 0-20 |  | -8.00 | 14707 | < .001 | 0.13 |
|  |  |  | 0.01 | 0.98 | - |  | -0.08 | 1.10 | - |  | -2.00 | 1097 | 0.02 | 0.09 |
|  | | | N |  |  |  | N |  |  |  |  |  |  |  |
| Sex (female) | | | 3772 |  |  |  | 3567 |  |  |  | 6.50 | 1 | .011 | - |
| *Note*. *SD* = standard deviation; *t* = independent t-test; *df* = degrees of freedom;  *chi^2^*= chi square test*; d/h* = Cohen’s effect sizes; Conduct disorder symptoms were assessed via the Developmental and Well-being Assessment (DAWBA) interview at age 16; Impulsivity was assessed via the DAWBA at age 13. CU traits = Callous unemotional traits; assessed at age 13 from the ALSPAC Wellbeing of my Teenage Son/Daughter questionnaire whereby certain items reflect the six subscale of CU traits. Family adversity was assessed between birth to age 4 assessed via the Family Adversity Index (FAI). Substance use was assessed at age 13 via the adapted version Structured Assessment for the Genetics of Alcoholism. Scores were included in a principal component analysis with varimax rotation to obtain an overall composite score reflecting substance use. | | | | | | | | | | | | | | |
|  |  |  |  |  |  |  |  |  |  |  |  |  |  |  |
|  |  |  |  |  |  |  |  |  |  |  |  |  |  |  |
|  |  |  |  |  |  |  |  |  |  |  |  |  |  |  |

| **ALSPAC “Teen Focus 2” clinic interview** | **Semi-Structured Assessment for the Genetics of Alcoholism (SSAGA)** |
| --- | --- |
| **Alcohol** | |
| Have you ever drunk alcohol like beer, cider, wine or spirits without your parents’ permission? (Y/N) | Now I would like to ask you some questions about your use of alcoholic beverages, like beer, wine, wine coolers, champagne, or hard liquor like vodka, gin, or whiskey. Have you ever had a drink of alcohol? (Y?N) |
| If yes: **How many times in the last 6 months** | We would like to know the number of alcoholic drinks you've had each day in the last week, and how long it took you to drink them.  Today is ___(**DAY OF THE WEEK**) Lets begin with yesterday  How many drinks of (beer, wine, liquor) did you have on **(NAME DAY OF WEEK)?** |
| **Cigarette smoking** | |
| Have you ever smoked cigarettes? (Y/N) | Now I’m going to ask you some questions about using tobacco. Have you ever smoked cigarettes daily for a month or more? (Y/N) |
| If yes: **How many times in the last 6 months?** | For how long (months) did you smoke/use tobacco? |
| **Cannabis use** | |
| Have you ever tried cannabis? (Y/N) | Have you ever used marijuana or hashish? (Y/N) |
| If yes: **How many times in the last 6 months?** | If yes: How many times? |

*Note*. The present study only utilised questions on frequency of engagement with substance use (bold questions in ALSPAC column). Responses to items were categorised by ALSPAC team as Nil, 1 per week, 1-3 times, > 4 times. The researchers did not make any additional adaptations for this study.

**Supplementary Table 2**. Questions from ALSPAC “Teen Focus 2” clinic and the Semi-Structured Assessment for the Genetics of Alcoholism (SSAGA)
